# Severity of anhedonia is associated with hyper-synchronization of the salience-default mode network in non-clinical individuals: a resting state EEG connectivity study

**DOI:** 10.1007/s00702-025-02894-3

**Published:** 2025-02-15

**Authors:** Claudio Imperatori, Giorgia Allegrini, Aurelia Lo Presti, Giuseppe A. Carbone, Mauro Adenzato, Benedetto Farina, Rita B. Ardito

**Affiliations:** 1 Experimental and Applied Psychology Laboratory, Department of Human Sciences, European University of Rome, Rome, Italy; 2 Department of Psychology, University of Turin, Turin, Italy

**Keywords:** Anhedonia, Triple Network, General psychopathology, Functional connectivity, EEG, eLORETA

## Abstract

Anhedonia is a core transnosographic symptom in several neuropsychiatric disorders. Recently, the Triple Network (TN) model has been proposed as a useful neurophysiological paradigm for conceptualizing anhedonia, providing new insights to clinicians and researchers. Despite this, the relationship between the functional dynamics of TN and the severity of anhedonia has been relatively understudied in non-clinical samples, especially in the resting state (RS) condition. Therefore, in the current study, we investigated this relationship using electroencephalography (EEG) functional connectivity. Eighty-two participants (36 males; mean age: 24.28 ± 7.35 years) underwent RS EEG recording with eyes-closed and completed the Beck Depression Inventory-derived 4-item anhedonia scale (BDI-Anh4) and the Brief Symptoms Inventory (BSI). EEG data on functional connectivity were analyzed using the exact low-resolution electromagnetic tomography (eLORETA). A significant positive correlation was observed between the BDI-Anh4 total score and salience-default mode network connectivity in the beta frequency band (*r* = 0.409; *p* = 0.010). The results of the hierarchical linear regression analysis also showed that this connectivity pattern was positively and independently associated (*β* = 0.358; *p* < 0.001) with the BDI-Anh4 total score and explained an additional 11% of the anhedonia variability. The association between anhedonia severity and increased salience-default mode network synchronization detected in the current study may reflect difficulty disengaging from internal/self-related mental contents, which consequently impairs the processing of other stimuli, including rewarding stimuli.

**Supplementary Information:**

The online version contains supplementary material available at 10.1007/s00702-025-02894-3.

## Introduction

Anhedonia (i.e., the loss of pleasure or interest in all or almost all activities) is a core transnosographic symptom in numerous neuropsychiatric disorders, particularly major depressive disorder (MDD) and schizophrenia (SZ) (Guineau et al. 2023; Trøstheim et al. 2020). It is associated with relevant clinical sequelae, including increased illness severity, chronicity, and poor response to treatment (Craske et al. 2014; Der-Avakian and Markou 2012; McMakin et al. 2012; Morris et al. 2009; Winer et al. 2014). Importantly, anhedonia is also detected dimensionally in non-clinical samples (Chan et al. 2012; Franken et al. 2007; Tobe et al. 2023). For example, a recent meta-analysis of adults with and without mental illness (Trøstheim et al. 2020) showed that although clinical samples, particularly patients with MDD, reported higher levels of anhedonia across multiple pleasure domains than healthy individuals, an estimated 14% of the adult non-clinical population scored above the Snaith-Hamilton Pleasure Scale cutoff (Snaith et al. 1995).

In recent years, the use of neuroimaging techniques such as functional magnetic resonance imaging (fMRI), electroencephalogram (EEG), and positron emission tomography (PET), has consistently improved our knowledge of the neurophysiological basis of this psychiatric symptom by identifying several alterations in brain circuitry and chemical dysregulations, particularly in the mesolimbic dopamine pathway (Borsini et al. 2020; Der-Avakian and Markou 2012; Wang et al. 2021).

More recently, the Triple Network (TN) paradigm (Menon 2011) has been proposed as a useful neurophysiological framework for conceptualizing anhedonia, providing new insights to clinicians and researchers (Pisoni et al. 2021). This model explains how three major brain networks, namely the default mode network (DMN), the central executive network (CEN), and the salience network (SN), work in an integrated manner to regulate arousal, attention, and cognitive abilities properly (Menon 2011). In particular, studies on clinical samples (Pisoni et al. 2021) have shown that high levels of anhedonia are associated with various aberrant intra- and inter-connectivity patterns, especially across SN nodes such as the anterior insula (AI). For example, compared to healthy controls, patients showed excessive connectivity of the SN in the resting state (RS) condition as well as SN abnormal activity and/or connectivity in response to emotional stimuli (e.g., decreased SN-DMN connectivity in response to positive stimuli and increased SN activity in response to negative stimuli) (Pisoni et al. 2021). FMRI-studies in individuals without psychopathology also appear to confirm the role of the SN in modulating cognition when hedonic rewards are present. For example, in non-clinical samples, regardless of stimulus valence, greater hedonic capacity has been reported to be associated with increased activation of several SN nodes (e.g., the AI) (Pisoni et al. 2021). More specifically, the SN is critically involved in detecting the presence of salient stimuli, including rewards, and in controlling the dynamic switch between internal/self-related and external/environment-related mental contents (i.e., the switch between DMN and CEN activity; Bolton et al. 2020; Sridharan et al. 2008). Therefore, the TN alterations observed in clinical populations may suggest that anhedonia is mainly related to deficits in saliency-mapping (Pisoni et al. 2021).

Although the relationship between TN connectivity and anhedonia has been recently investigated in patients with various mental disorders, the RS dynamic interactions among SN, DMN, and CEN are relatively unexplored in non-clinical samples, especially by EEG. Among brain imaging techniques, EEG is not only considered a valid tool for assessing brain network interactions (Liu et al. 2018) but also an affordable and valuable tool for analyzing and understanding the electrophysiological properties underlying anhedonia-related processes due to its excellent temporal resolution (Gupta et al. 2024; Keren et al. 2018; Sun et al. 2023). Furthermore, compared to other neuroimaging methods, the EEG connectivity analysis, investigating how brain areas interact with each other in different frequency bands, has the important advantage in detecting the specific electrophysiological signature that characterizes neural networks (Mantini et al. 2007; Whitton et al. 2018). This is particularly relevant for the RS condition, which reflects the organization of intrinsic brain activity and provides valuable information about the link between the dynamic organization of multiple neural circuits and cognition, emotion, and behavior (Anderson and Perone 2018; Blanken et al. 2021; Deco et al. 2011).

In light of this, and to further elucidate the complex relationships between the dynamics of large-scale RS networks and anhedonia, we investigated this relationship in a non-clinical sample within the TN framework. Investigating anhedonia in non-clinical individuals (i.e., individuals who do not already have diagnosed psychiatric disorders) from the perspective of large-scale brain networks may play an important role in understanding the physio-pathogenic mechanisms related to the onset and progression of various mental disorders such as SZ and MDD (Harvey et al. 2007; Keller et al. 2013). Furthermore, investigating the neurophysiological basis of distinct psychopathological dimensions unrelated to overt clinical pictures may be useful in providing specific and tailored treatment plans (Luyten and Fonagy 2018; Maj 2016).

According to the saliency-mapping deficit model (Pisoni et al. 2021), we hypothesized that high levels of anhedonia would be associated with increased RS-EEG connectivity between SN nodes.

## Materials and methods

### Participants and procedure

An a priori power analysis was performed using G*Power 3.1. software (Faul et al. 2009) considering a statistical power of 1 – β = 80%, an α-error probability of 0.05, and an effect size of *r* = 0.30. According to the software, a sample of 82 participants was required in a two-sided test correlation model to achieve satisfactory statistical power.

Recruitment lasted from June 2023 to February 2024. Inclusion criteria were: (i) age ≥ 18 years; (ii) Italian native language; (iii) providing formal informed consent. Exclusion criteria were: (i) current and/or previous self-reported psychiatric or neurological disorders; (ii) left-handedness [i.e., Laterality Quotient < 61 (Veale 2014)]; (iii) self-reported use of drugs and psychoactive substances in the last two weeks prior to the EEG recording.

Participants were recruited via an online survey conducted using web-based tools (e.g., social networks, emails, mailing lists, instant messaging) to assess inclusion/exclusion criteria. Eighty-two participants (36 males and 46 females; mean age: 24.28 ± 7.35 years) met the inclusion criteria and were finally included in the current study. Thus, these participants completed a second online Google form that included the Beck Depression Inventory-derived 4-item anhedonia scale (BDI-Anh4; Beck et al. 1996) and the Brief Symptoms Inventory (BSI; Derogatis and Melisaratos 1983). In addition, socio-demographic variables (e.g., biological sex, age, and education level), frequency of alcohol consumption as assessed by the first item (i.e., “How often do you have a drink containing alcohol?”) of the Alcohol Use Disorders Identification Test (AUDIT#1; Babor et al. 2001), and tobacco use data were collected. In the following week, participants performed the EEG recording. In accordance with the principles of the Helsinki Declaration, the ethics review committee of the University of Turin reviewed and approved the present study (protocol n. 0243029). All participants took part in this study voluntarily (i.e., they did not receive any payment or academic credits).

The current study is part of a larger research project on the relationship between EEG data and psychopathology in the general population; therefore, the included sample partially overlaps with the sample of two manuscripts previously published by our research group (Carbone et al. 2024a, b).

## Questionnaires

The BDI-Anh4 is a commonly used scale to assess the level of anhedonia (Cogan et al. 2024). It consists of 4 items (i.e., “*loss of pleasure*”, “*loss of interest*”, “*loss of energy*”, and “*loss of interest in sex*”) that were originally included in the BDI (Beck et al. 1996). Each item is scored on a 4-point scale (from 0 to 3), with higher scores reflecting higher levels of anhedonia. The scale is characterized by appropriate psychometric properties, including satisfactory convergent and discriminant validity (Cogan et al. 2024). In the present sample, the Italian version of the BDI-Anh4 was used (adapted from Sica and Ghisi 2007), and Cronbach’s alpha was 0.63.

The BSI (Derogatis and Melisaratos 1983) is a self-report measure commonly used to assess general psychopathology (Ryan 2007). It is a 53-item questionnaire that examines the following psychopathological dimensions: somatization, obsession-compulsion, interpersonal sensitivity, depression, hostility, anxiety, phobic anxiety, paranoid ideation, and psychoticism. Respondents were asked to rate each item on a 5-point Likert scale (0–4), with higher scores indicating more severe psychopathological symptoms. The measure of the level of general psychopathology was determined using the General Severity Index (GSI), which is the mean of all subscale scores. In the present study the Italian version of the BSI was used (Leo et al. 1993), and Cronbach’s alpha for the GSI was 0.96.

## EEG recording and analysis

RS EEG recordings with eyes-closed were performed in an EEG laboratory using 62 active electrodes with ground and reference placed at electrode positions AFz and FCz, respectively (BrainAmp DC from Brain Products). The EEG headset montage was performed according to the 10–20 system (see supplementary Fig. 1 for electrode sites), and the impedances were kept below 5 kΩ. Each EEG recording lasted 5 min, with participants seated in a quiet and silent semi-darkened room (Massullo et al. 2020). Subjects were instructed to abstain from alcohol and/or caffeine products for at least 4 h prior to the EEG recording.

Artifacts rejection was performed using the EEGLAB toolbox for MATLAB (version 2022.1; Delorme and Makeig 2004). After an initial visual inspection and removal of the most evident artifacts (e.g., muscle movements), a passband filter of 1–40 Hz was used, and average re-reference was applied. Finally, using the infomax decomposition algorithm, an Independent Component Analysis (ICA) was performed to remove specific artifacts (e.g., electrical artifacts). Full details about EEG data processing are previously reported (Imperatori et al. 2023).

After the raw data processing, connectivity analysis was performed using the Exact Low-Resolution Electromagnetic Tomography software (eLORETA; Pascual-Marqui et al. 2011). The eLORETA is considered a well-validated device for the localization of electrocortical activity (Asadzadeh et al. 2020; Halder et al. 2019; Jatoi et al. 2014) and for the assessment of large-scale brain networks (Liu et al. 2018). By using a three-dimensional (3D), linear, distributed non-inverse norm solution, the software guarantees an accurate reconstruction of the bioelectric source, despite the low spatial resolution (Canuet et al. 2012). In particular, the application of the boundary electric method (BEM) for the potential electric field computation improves the localization accuracy by replacing the standard spherical head model with a standardized realistic head model (Fuchs et al. 2002). This model is based on an averaged magnetic resonance images (MRI) data set comprising 152 templates provided by the Brain Imaging Center of the Montréal Neurological Institute (MNI; Mazziotta et al. 2001).

In the current study, to assess connectivity within the TN, nine regions of interest (ROIs; Table 1) were pre-defined according to previous eLORETA reports (Carbone et al. 2022; Filosa et al. 2024; Imperatori et al. 2020; Massullo et al. 2022) using the “ROI-maker#1 method” available in the software (i.e., based on the MNI coordinates). In line with previous studies (e.g., Canuet et al. 2011; de la Salle et al. 2016; Kitaura et al. 2017), the “single nearest voxel” option (i.e., each ROI consisting of a single voxel, the closest to each TN hub), implemented in the eLORETA package, was selected to perform the source reconstruction of the ROIs.

**Table 1:** The eLORETA source reconstruction Triple Network

Brain networks	eLORETA MNI coordinates			Brain structure
	x	y	z	
DMN	0	55	25	medial PFC
	0	-55	20	PCC
CEN				
	-45	20	35	left DLPFC
	40	25	50	right DLPFC
	-40	-70	45	left PPC
	50	-60	40	right PPC
SN				
	0	20	35	dorsal ACC
	-45	15	-5	left AI
	50	15	-5	right AI

*Abbreviation* eLORETA = Exact Low-Resolution Electromagnetic Tomography software; MNI = Montréal Neurological Institute; DMN = Default Mode Network; CEN = Central Executive Network; SN = Salience Network; PFC = Prefrontal Cortex; PCC = Posterior Cingulate Cortex; DLPFC = Dorsolateral Prefrontal Cortex; PPC = Posterior Parietal Cortex; ACC = Anterior Cingulate Cortex; AI = Anterior Insula

In accordance with the guidelines proposed by Miljevic and colleagues (2022), the post-processed EEG data (i.e., artifacts free files) were divided into 4-second epochs, and the lagged phase synchronization (LPS) algorithm (Pascual-Marqui et al. 2011) was used to calculate synchronization within TN. This index is one of the most commonly used for the assessment of functional EEG connectivity as it reduces artifacts by removing the instantaneous zero-lag contribution (Hata et al. 2016; Olbrich et al. 2014). Using the normalized Fourier transform, the LPS measures the signal similarity in the frequency domain, with values ranging from 0 (i.e., absence of synchronization) to 1 (i.e., extreme synchronization). In the current study, the LPS was investigated in the following frequency bands: delta (1–4 Hz), theta (4.5–7.5 Hz), alpha (8–13 Hz), beta (13.5–30 Hz).

Suitable EEG recordings for connectivity analysis were acquired for all study participants. Qualitative visual inspection of the EEG signal showed no relevant evidence of abnormal patterns or signs of sleepiness during the RS recordings.

### Statistical analysis

As a primary analysis, the relationship between the BDI-Anh4 total score and the TN connectivity data was examined using the regression analysis option available in the eLORETA software (i.e., the connectivity values between each ROI, in each frequency band, were correlated with the BDI-Anh4 total score). This analysis was performed using the statistical non-parametric mapping method (Nichols and Holmes 2002). This technique involves the computation of 5000 randomizations to determine critical probability thresholds for the *r-*values and the corresponding statistically corrected *p*-values. The statistical non-parametric mapping method is considered a valid tool for solving the multiple testing problem because it controls multiple comparisons among all connections in each frequency band without the need to rely on Gaussianity (for technical details, see Hata et al. 2016).

To investigate the unique contribution of TN connectivity data to anhedonia, a hierarchical regression analysis was performed as a complementary analysis with the BDI-Anh4 total score as the dependent variable. More specifically, in the first step, sociodemographic data (i.e., sex, age, education level, profession, and marital status), tobacco and alcohol use, and the level of general psychopathology (i.e., GSI-BSI score) were included in the model. In the second and final block, each significant TN connectivity data found in the primary analysis was included in the equation. In each block, all independent variables were entered into the model simultaneously, using the “standard method of entry”. Multiple regression assumptions were tested in accordance with Williams and colleagues (2013). The normality of the data was checked according to Kim and colleagues (2013), while multicollinearity was analyzed by calculating the tolerance value and the Variance Inflation Factor (VIF) for each variable. Influential data points were then determined using Cook’s distances. Results were presented as standardized beta (*β*) coefficients and corresponding 95% confidence intervals (CI). All additional analyses were performed using SPSS version 26.0 (IBM, Armonk, NY, USA).

## Results

In the current sample, the mean BDI-Anh4 total score was 2.56 ± 2.03. The other descriptive statistics are shown in Table 2.

**Table 2:** Descriptive statistic for the sample (*N* = 82). **Variables**

Age – M ± SD		24.28 ± 7.35
Female – N (%)		46 (56.1%)
Education years – M ± SD		15.82 ± 1.77
Students – N (%)		19 (23.2%)
Married/or living with a Partner – N (%)		4 (4.9%)
Tobacco use – N (%)		33 (40.2%)
Alcohol use		
Never – N (%)		4 (4.9%)
Monthly or less – N (%)		18 (22.0%)
2–4 times a month – N (%)		37 (45.1%)
2–3 times a week – N (%)		20 (24.4%)
4 or more times a week – N (%)		3 (3.7%)
GSI-BSI – M ± SD		1.13 ± 0.64
BDI-Anh4 – M ± SD		2.56 ± 2.03

*Abbreviation* M = mean; SD = standard deviation; BDI-Anh4 = the Beck Depression Inventory-derived 4-item anhedonia scale; GSI-BSI = Global Severity Index of the Brief Symptoms Inventory

**Fig. 1:**
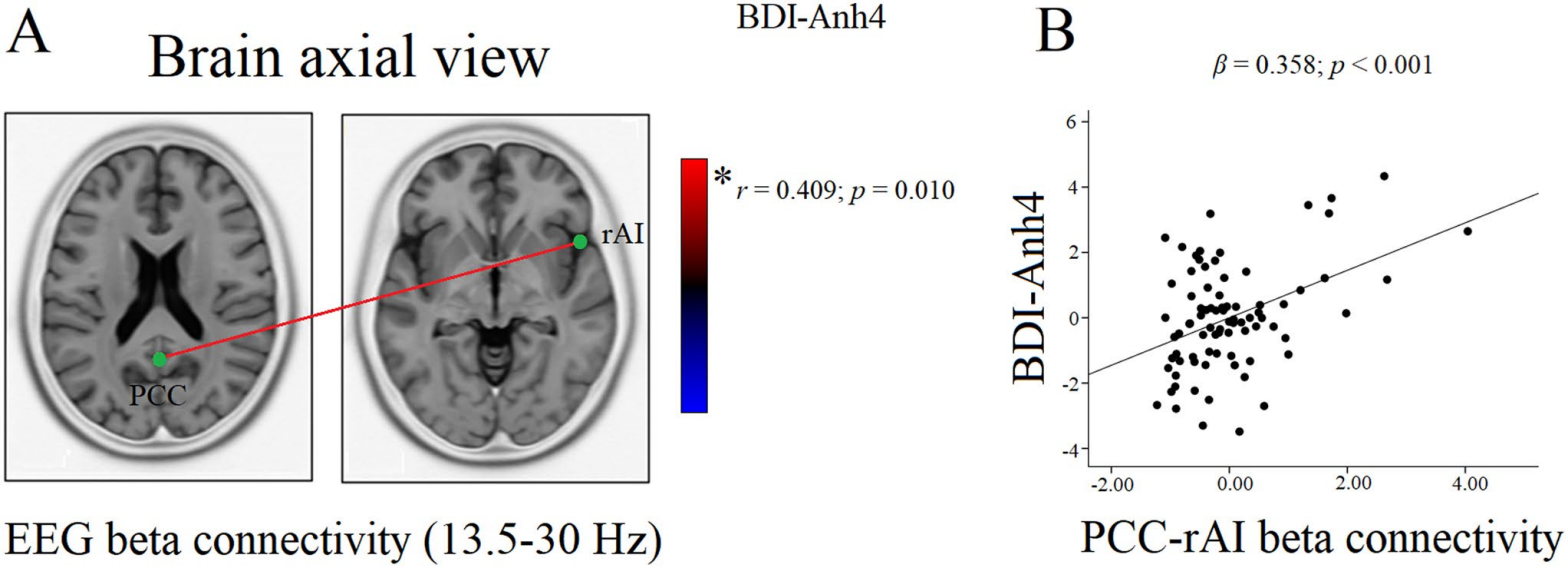
**Panel A**. Significant correlation between PCC-rAI connectivity and BDI-Anh4 total score in the beta frequency band. Red lines show a positive correlation between the severity of anhedonia and the strength of connections between two interconnected ROIs. Blue lines (not present) would indicate a negative correlation between the severity of anhedonia and the strength of connections between two interconnected ROIs. Figure 1B. Scatterplot of the relationship between PCC-rAI connectivity values and BDI-Anh4 total score (values are adjusted for sex, age, education level, profession, marital status, tobacco and alcohol use, GSI-BSI). *Abbreviation* PCC = posterior cingulate cortex; rAI = right anterior insula; BDI-Anh4 = the Beck Depression Inventory-derived 4-item anhedonia scale; ROIs = Regions of Interest; GSI-BSI = Global Severity Index of the Brief Symptoms Inventory

In the current study, the average epochs analyzed were 74.45 ± 7.68. The eLORETA thresholds for statistical significance (i.e., corrected for multiple comparisons) were *r* = ± 0.410 (corresponding to *p* = 0.01) and *r* = ± 0.366 (corresponding to *p* = 0.05). A significant positive correlation between the BDI-Anh4 total score and SN-DMN connectivity was observed in the beta frequency band (*r* = 0.409; *p* = 0.010). More specifically, higher levels of anhedonia were associated with increased beta connectivity between the posterior cingulate cortex (PCC) and the right AI (Fig. 1A). No significant correlations were found in the other frequency bands.

The results of the hierarchical linear regression analysis are shown in Table 3. All linear regression assumptions were satisfied, including heteroscedasticity (i.e., Breusch-Pagan test F_9;72_ = 0.761; *p* = 0.652). The tolerance and VIF statistics indicated that there was no relevant multicollinearity problem (i.e., tolerance values > 0.10 and VIF < 5), and Cook’s distances were also adequate (i.e., maximum value = 0.093). The models explained between 30% and 41% of the variability in the BDI-Anh4 total score. In the final block, when controlling for the presence of other variables, beta connectivity values were positively and independently associated with BDI-Anh4 total score (*β* = 0.358; *p* < 0.001) and explained an additional 11% of anhedonia variability; thus, increased RS SN-DMN functional connectivity was associated with higher levels of anhedonia when controlling for potential confounding variables (Fig. 1B). In the final block, BSI-GSI was also positively and independently associated with BDI-Anh4 total score (*β* = 0.497; *p* < 0.001).

**Table 3:** Hierarchical linear regression analysis (*N* = 82)

Dependent Variable	Adjusted *R*^2^	F	Block		*R*^2^ Change	F Change	Independent Variable	β	95% CI	*p*
BDI-Anh4 Total score	0.297	5.283^1^*		1	0.367*	5.283*	Age	0.231	[0.004; 0.124]	0.037
							Sex	0.081	[-0.471; 1.128]	0.415
							Profession	0.083	[-0.722; 1.513]	0.483
							Education	-0.026	[-0.259; 0.199]	0.793
							Marital status	-0.074	[-2.492; 1.109]	0.446
							Tobacco use	0.134	[-0.313; 1.416]	0.208
							AUDIT#1	0.025	[-0.445; 0.556]	0.826
							GSI-BSI	0.521	[0.977; 2.183]	< 0.001
	0.414	7.369^2^*		2	0.113*	15.600*	Age	0.176	[-0.007; 0.104]	0.084
							Sex	0.004	[-0.731; 0.762]	0.966
							Profession	0.002	[-1.031; 1.047]	0.988
							Education	-0.022	[-0.234; 0.184]	0.810
							Marital status	-0.063	[-2.237; 1.052]	0.475
							Tobacco use	0.185	[-0.035; 1.558]	0.061
							AUDIT#1	-0.008	[-0.477; 0.440]	0.936
							GSI-BSI	0.497	[0.977, 2.183]	< 0.001
							PCC-right AI beta connectivity*	0.358	[0.360; 1.094]	< 0.001

Note: * *p* < 0.001; * = connectivity data are expressed as standardized values; DF = ^1^8:73; ^2^9:72

*Abbreviation*: BDI-Anh4 = the Beck Depression Inventory-derived 4-item anhedonia scale; AUDIT#1 = first item of the Alcohol Use Disorders Identification Test; GSI-BSI = Global Severity Index of the Brief Symptoms Inventory; PCC = posterior cingulate cortex; AI = anterior insula.

## Discussion

In the current study, we investigated the relationship between anhedonia and the functional dynamics of RS-TN in a non-clinical sample. Our results showed that higher levels of anhedonia were associated with increased inter-network functional connectivity in the TN. More specifically, the BDI-Anh4 total score was positively correlated with increased beta EEG coherence between the SN (in the right AI) and the DMN (in the PCC). Our regression analysis also revealed that this connectivity pattern was positively and independently associated with the severity of anhedonia after controlling for the presence of other potential confounding factors (e.g., the general level of psychopathology), suggesting that an increased interaction between the SN and the DMN in beta frequency may be considered a relevant factor in the conceptualization of this psychopathological symptom.

To our knowledge, this is the first study to investigate the relationship between functional RS-EEG connectivity within TN and the severity of anhedonia in a non-clinical sample. The current data are consistent with previous reports suggesting that the TN model is a suitable framework for understanding mechanisms related to anhedonia (Pisoni et al. 2021) and highlighting, as hypothesized, the role of altered saliency-mapping in the physiopathology of this clinical dimension.

In particular, our results seem to suggest that high levels of anhedonia at rest are electrophysiologically characterized by increased communication between the SN and the DMN. Indeed, increased beta connectivity has been conceptualized as an electrophysiological signature of hyperexcitability (Lee et al. 2021; Park et al. 2018), and previous studies linked beta oscillations and synchronization to motivational processes and salience circuits (Martinez-Maldonado et al. 2020; Schimmelpfennig et al. 2023). The SN is considered a task-positive neural system that is significantly involved in the detection of relevant and salient stimuli (Uddin 2016). Among the SN hubs, the right AI plays a crucial role in switching between DMN-related self-referential processes and CEN-related goal-directed cognitive activities (Sridharan et al. 2008). Specifically, the DMN is the brain circuit that is most active during the RS condition and is involved in a variety of self-related mental processes, such as self-consciousness, mind-wandering, and autobiographical memory (Andrews-Hanna 2012; Vicari and Adenzato 2014). Of relevance, it is also known that these self-related activities are mainly driven by the engagement of the PCC (Davey et al. 2016).

With this in mind, the positive association between the BDI-Anh4 total score and the increased SN-DMN beta connectivity found in the current study could be the result of a difficulty in disengaging from internal/self-related mental contents, thereby impairing the processing of other stimuli, including rewarding stimuli. In other words, hyper-synchronization between SN and DMN during RS might reflect the tendency of individuals with high levels of anhedonia “*to be pre-formed for self-referential processing*” at the expense of emotional and hedonic stimuli (Koeppel et al. 2021). Accordingly, a recent study showed that adolescents with anhedonia had higher frequency of mind-wandering, especially to unpleasant topics, compared to controls, and that this tendency was associated with stronger SN-DMN connectivity.

Intriguingly, it is well known that AI dysfunction within TN can contribute to abnormal inter-network switching, leading to dysfunctional behavioral responses to both internal and external stimuli (Menon and Uddin 2010). In particular, altered SN-DMN connectivity is one of the most consistent neurophysiological alteration found in clinical conditions characterized by high levels of anhedonia, such as SZ and MDD (Macedo et al. 2022; Mulders et al. 2015; Sha et al. 2019). In this context, it has been suggested that aberrant SN-DMN connectivity may contribute to depression development/maintenance, possibly explaining patients’ difficulties in disengaging from self-focused mental contents, often negatively biased thoughts (Manoliu et al. 2013).

According to a dimensional clinical perspective, in order to characterize more specifically the symptomatology of different patients and to design more targeted interventions, it is important to study the individual psychopathogenic components, and their related neurophysiological patterns that contribute to the different clinical pictures (Luyten and Fonagy 2018; Maj 2016; Stein et al. 2024). For example, it is known that patients with MDD and high levels of anhedonia are more likely to have a worse prognosis, including deficits in physical, psychological, and social functioning, and require specific therapeutic tools (Wong et al. 2024). In this context, the administration of drugs with specific effects against anhedonia, such as ketamine (Patarroyo-Rodriguez et al. 2024), and/or the use of non-invasive brain stimulation techniques, which have been shown to be effective specifically against this symptom (Chu et al. 2024), may be particularly useful.

Although the current findings may be of interest, several limitations should be considered. First, this is a cross-sectional report. Therefore, it is not possible to determine a causal relationship between the study variables and the path of such causality. In addition, although we controlled for the general level of psychopathology (i.e., GSI-BSI total score) and subjects with self-reported past and/or current mental diseases were not included in this study, a full clinical interview was not conducted; thus, it is possible that covert psychopathological conditions were not recognized. Third, although the eLORETA is a suitable software for assessing brain connectivity, its major limitation is its low spatial resolution, particularly in localizing subcortical areas (e.g., the striatum) known to be involved in anhedonia-related processes (Der-Avakian and Markou 2012). Fourth, the current study focused on the RS condition, so TN connectivity patterns in relation to rewarding stimuli were not exanimated, making our results specific to the RS condition with eyes closed. Therefore, further longitudinal studies conducted during both the RS and reward tasks should be implemented to better understand the TN neurophysiological basis of anhedonia in non-clinical samples.

Despite these limitations, our findings lend more specificity to this area of investigation, as most previous studies have investigated the relationship between anhedonia and TN dynamics in clinical samples or in healthy participants during emotional tasks (Pisoni et al. 2021), whereas here we examined this relationship during RS in a non-clinical sample. Studying non-clinical populations allows for the investigation of brain dynamics associated with psychopathological vulnerability without being altered by medication use or chronicity of the disorders. This represents an important source of information for researchers and clinicians on the neurophysiological mechanisms related to the onset and progression of a variety of psychiatric disorders (Marques et al. 2021).

Overall, our findings suggest that high levels of anhedonia are associated with increased synchronization between the SN and the DMN. This neurophysiological pattern may reflect the difficulty to disengage from internal/self-related mental contents, which consequently impairs the processing of environmental stimuli, including rewarding stimuli. Investigating the interaction between large-scale brain networks and anhedonia in non-clinical samples may play an important role in understanding the physio-pathogenic mechanisms associated with the onset and progression of specific clinical conditions characterized by high levels of anhedonia, such as SCZ and MDD.

## Electronic supplementary material

Below is the link to the electronic supplementary material.

Supplementary Material 1
