# Supplementary figures and images for: Severity of anhedonia is associated with hyper-synchronization of the salience-default mode network in non-clinical individuals: a resting state EEG connectivity study
Source: J Neural Transm (Vienna). 2025 Feb 15;132(5):731–41. doi: 10.1007/s00702-025-02894-3 (PMC12043527; doi:10.1007/s00702-025-02894-3)

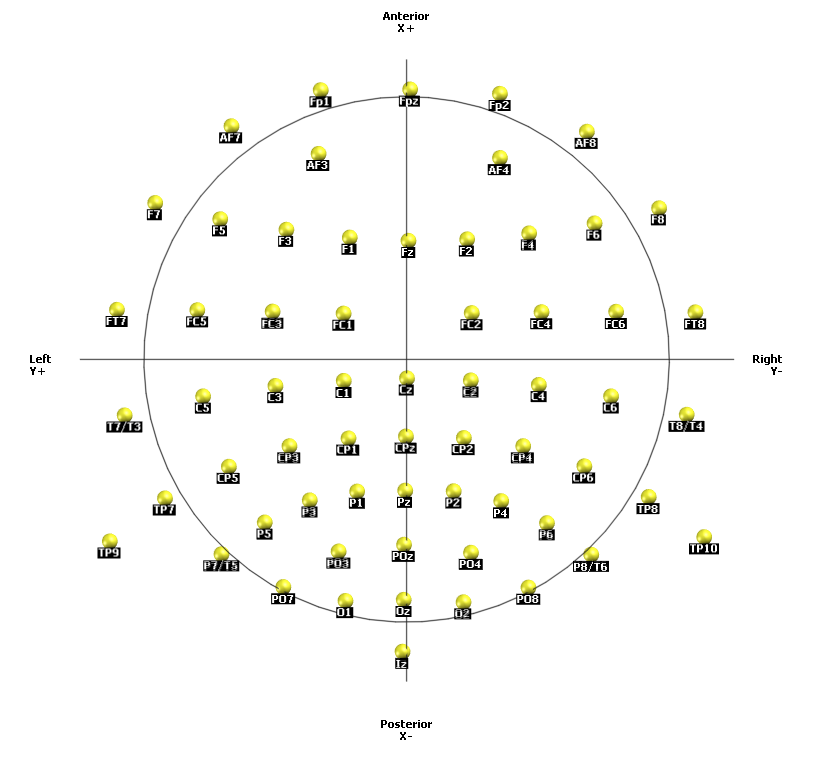

Supplement: Supplementary file 1 — Supplementary Material 1 [file 702_2025_2894_MOESM1_ESM.tif]
